# Abnormal hematopoietic phenotypes in Pim kinase triple knockout mice

**DOI:** 10.1186/1756-8722-6-12

**Published:** 2013-01-29

**Authors:** Ningfei An, Andrew S Kraft, Yubin Kang

**Affiliations:** 1 Division of Hematology-Oncology, Department of Medicine, Medical University of South Carolina, 86 Jonathan Lucas Street, Hollings Cancer Center Rm# HO307, Charleston, SC 29425, USA

**Keywords:** Serine/threonine kinase, Pim kinase, Hematopoietic stem cells, Hematopoietic stem cell transplantation, Proliferation, Apoptosis, Knockout mouse

## Abstract

**Background:**

Pim (proviral insertion in murine lymphoma) kinases are a small family of constitutively active, highly conservative serine/threonine oncogenic kinases and have 3 members: Pim1, Pim2, and Pim3. Pim kinases are also implicated in the regulation of B- and T- cell responses to cytokines and hematopoietic growth factors. The roles of Pim kinases in the regulation of primitive hematopoietic stem cells (HSCs) are largely unknown.

**Methods:**

In the current study, Pim1^−/−^2^−/−^3^−/−^ triple knockout (TKO) mice were used to determine the role of Pim kinases in hematopoiesis. Peripheral blood hematological parameters were measured in Pim TKO mice and age-matched wild-type (WT) controls. Primary, secondary, and competitive transplantations were performed to assay the long-term repopulating HSCs in Pim TKO mice. In vivo BrdU incorporation assay and ex vivo Ki67 staining and caspase 3 labeling were performed to evaluate the proliferation and apoptosis of HSCs in Pim TKO mice.

**Results:**

Compared to age-matched WT controls, Pim TKO mice had lower peripheral blood platelet count and exhibited erythrocyte hypochromic microcytosis. The bone marrow cells from Pim TKO mice demonstrated decreased hematopoietic progenitor colony-forming ability. Importantly, Pim TKO bone marrow cells had significantly impaired capacity in rescuing lethally irradiated mice and reconstituting hematopoiesis in primary, secondary and competitive transplant models. In vivo BrdU incorporation in long-term HSCs was reduced in Pim TKO mice. Finally, cultured HSCs from Pim TKO mice showed reduced proliferation evaluated by Ki67 staining and higher rate of apoptosis via caspase 3 activation.

**Conclusions:**

Pim kinases are not only essential in the hematopoietic lineage cell development, but also important in HSC expansion, self-renewal, and long-term repopulation.

## Background

Pim1, Pim2 and Pim3 belong to a small family of serine/threonine protein kinases and are evolutionarily conserved in multicellular organisms. Pim1 and Pim2 were originally identified from cloning the retroviral integration sites in murine Moloney Leukemia virus (MuLV)-induced lymphomas [1,2]. Pim3 was identified through high throughput retroviral tagging in tumors of c-Myc transgenic mice deficient for Pim1 and Pim2 [3]. Although Pim kinase genes are located on different chromosomes, they encode proteins with a high degree of sequence homology [4,5]. Additionally, the functions and expression patterns of Pim kinases overlap significantly with each other [4,6,7]. For example, Pim3 can compensate for the loss of Pim1 and Pim2 in MuLV-induced lymphomagenesis [3]. EμMyc-EμPim2 double transgenic mice develop B cell lymphoid tumors similar to those seen in EμMyc-EμPim1 double transgenic mice [8,9]. Pim kinases are constitutively active and play an important role in tumor cell cycle regulation and in cancer cell survival [5].

Over the last two decades, several genetically modified mice were generated to facilitate the studies of the functional roles of Pim kinases. These animal models included Eμ-Pim1 transgenic mice [8], Pim1^−/−^ single knockout (KO) mice [10], Pim2^−/−^ single KO mice [11], and Pim1^−/−^2^−/−^3^−/−^ triple KO (TKO) mice [4]. Pim1^−/−^ and Pim2^−/−^ single KO mice do not show any anatomic or developmental defects likely in part due to the functional redundancy and overlap of Pim kinases. Pim single or triple KO mice are all viable and show subtle hematological changes such as anemia, erythrocyte microcytosis, reduced peripheral T- and B- cell numbers, and impaired T- and B- cell responses to IL-2, IL-3 and IL-7 stimulation [4,10]. Grundler, et al. [12] recently found that Pim1 was important in regulating the surface expression of CXCR4 chemokine receptor in hematopoietic stem cells (HSCs). Pim1 phosphorylates serine 339 of the intracellular domain of CXCR4, a site critical for CXCR4 recycling [12]. However, very little is known about the effects of Pim kinases on hematopoiesis and the roles of Pim kinases in the expansion and proliferation of primitive HSCs.

We recently reported a quantitative real-time PCR-based technique for determination of donor cell engraftment in a competitive murine transplantation model [13]. Our PCR method measures the Y chromosome specific gene, i.e., Zfy-1, and can be used for any strain of mouse transplantation models. In the current study, we performed serial transplant experiments and competitive transplant experiments to analyze the hematopoietic phenotypes of Pim TKO mice. We showed that Pim TKO HSCs are deficient in self-renewal and long-term repopulation. These defects are at least in part due to reduced cell proliferation and increased cell apoptosis in the most primitive HSC compartment in Pim TKO mice.

## Results

### Thrombocytopenia and erythrocyte hypochromic microcytosis in Pim TKO mice

**Figure 1:**
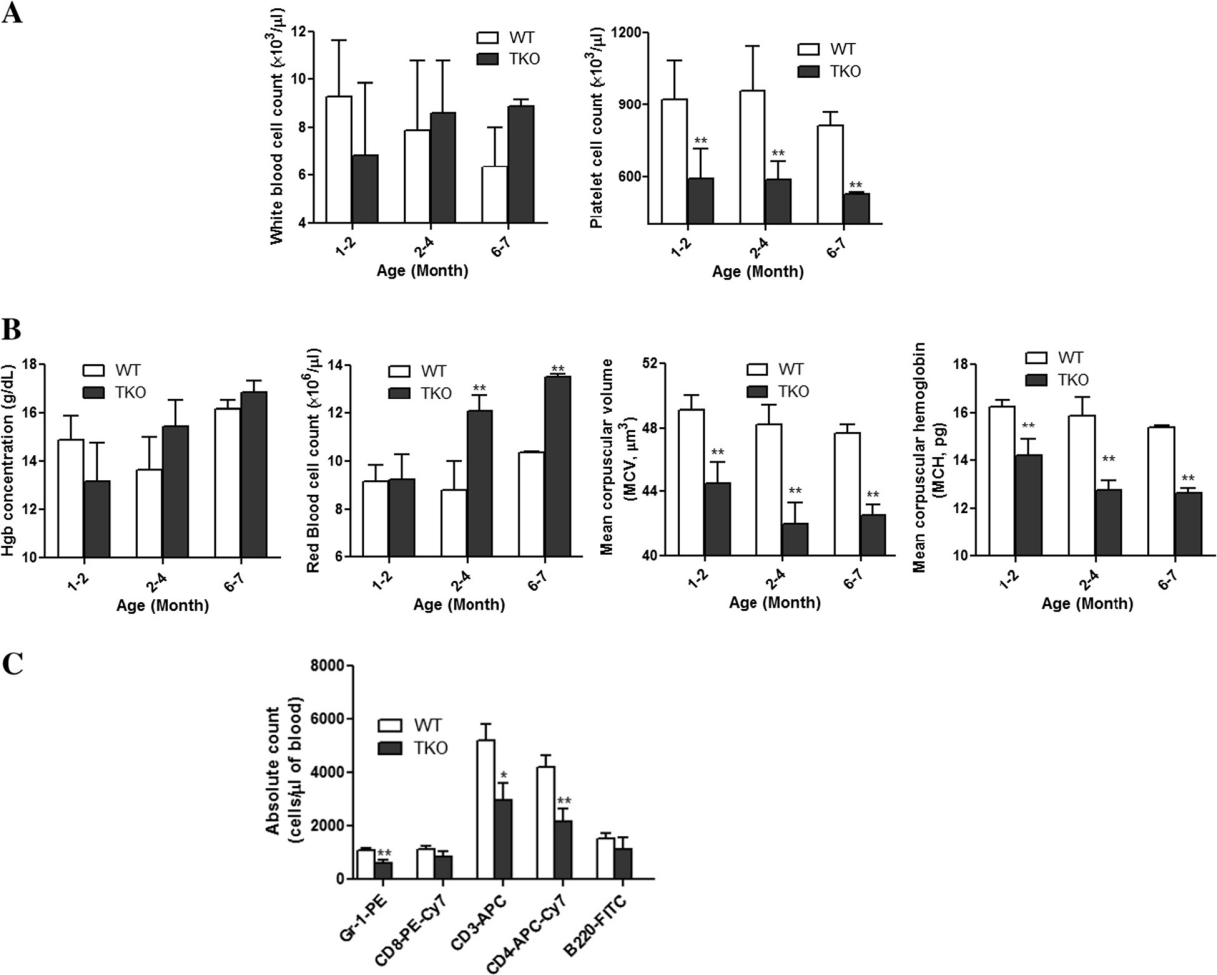
**Reduced peripheral platelet counts and erythrocyte hypochromic microcytosis in Pim TKO mice.** (**A**) Peripheral blood white blood cell count and platelet count. (**B**) Peripheral blood hemoglobin, red blood cell count, MCV and MCH. Peripheral blood samples were collected from 3 different age groups of Pim TKO mice and age-matched WT control mice. Peripheral hematological parameters were measured using Scil ABC plus hematology analyzer (** p < 0.01). Group 1: 1–2 months old (n = 8 for Pim TKO mice); Group 2: 2–4 months old (n = 8 for Pim TKO mice); Group 3: 6–7 months old (n = 3 for Pim TKO mice). (**C**) Peripheral blood cell subset analysis. Peripheral blood was obtained from 3 months old Pim TKO mice and age-matched WT controls, stained with various antibodies as described in the Materials and Methods, and analyzed by flow cytometry. (*p < 0.05, **p < 0.01).

To determine the effects of Pim kinases on hematopoiesis, we first measured peripheral blood white blood cells, red blood cells, platelets and hemoglobin in 3 different age groups of Pim TKO mice (*i*.*e*., 1–2 months old, 2–4 months old and 6–7 months old). Sex- and age- matched wildtype (WT) control mice were used for comparison. While the total white blood cell counts were comparable between Pim TKO mice and WT controls, the platelet count was significantly reduced in Pim TKO mice (Figure 1A, p < 0.01). Consistent with previous reports with Pim deficient mice [4,10,11], Pim TKO mice exhibited erythrocyte hypochromic microcytosis that was characterized by unchanged total hemoglobin level with increased red blood cell count (Figure 1B, 1^st^ and 2^nd^ panel). The erythrocyte hypochromic microcytosis was further confirmed by measuring the mean corpuscular volume (MCV) and the mean corpuscular hemoglobin (MCH) of red blood cells, both of which were significantly reduced in Pim TKO mice (Figure 1B, 3^rd^ and 4^th^ panel, p < 0.01). We then performed a detailed peripheral cell subset analysis that quantified the absolute number of peripheral Gr-1^+^ granulocytes, CD3^+^ T cells, CD4^+^ cells, CD8^+^ cells and B220^+^ B cells [14]. Compared to WT controls, Pim TKO mice had significantly lower number of CD3^+^ T cells, predominantly in the CD4^+^ T helper cell population (Figure 1C, p < 0.01). Gr-1^+^ granulocytes were also reduced in Pim TKO mice. These results suggested that Pim TKO mice had impairment in multiple lineages of hematopoietic cells.

### Reduced hematopoietic stem/progenitor cell number and colony-forming units in Pim TKO mice

**Figure 2:**
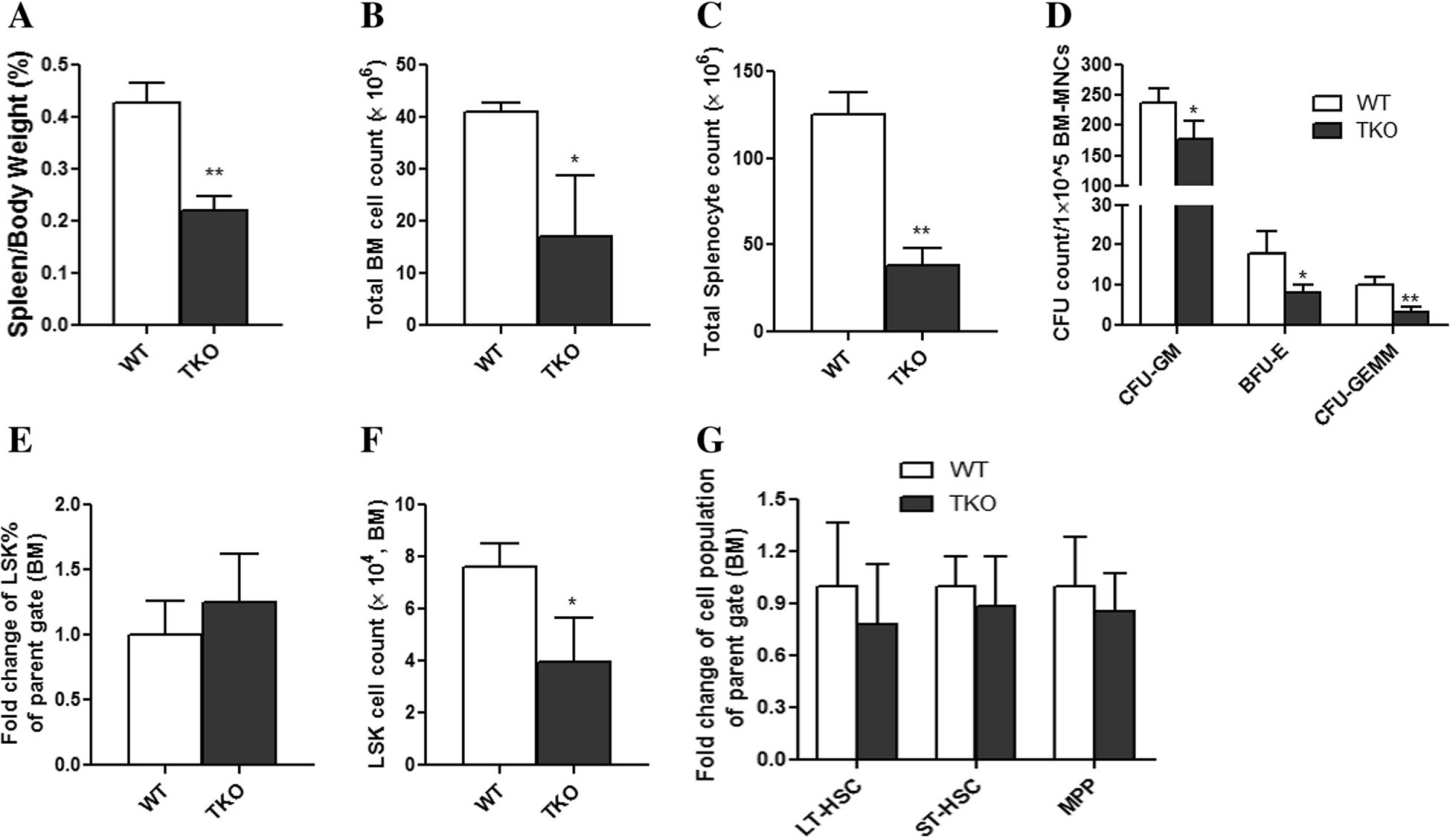
**Spleen weight, total BM cell number, and CFUs in Pim TKO mice.** (**A**) Reduced spleen weight in Pim TKO mice. Spleens from Pim TKO mice and WT controls were isolated and weighted. Data shown are the percentage of spleen weight to total body weight (n=5, **p < 0.01). (**B**) Reduced total bone marrow cells in Pim TKO mice. Bone marrow cells from 2 femurs and 2 tibias were harvested from Pim TKO mice and age-matched WT mice, depleted of RBCs, and counted (n=7, *p < 0.05). (**C**) Reduced total splenocytes in Pim TKO mice. Spleens from Pim TKO mice and age-matched WT mice were harvested, depleted of RBCs and counted (n=5, **p < 0.01). (**D**) Reduced CFUs in Pim TKO mice. BM cells were harvested from Pim TKO mice or WT controls and plated in Methocult^®^ GF 3434 (1x10^5^/dish in triplicate). The numbers of CFUs-GM, BFUs-E and CFUs-GEMM were counted at day 7, day 9, and day 12, respectively (n=4, *p < 0.05, **p<0.01). (**E**) Comparable percentages of LSK cells in Pim TKO mice. RBC-depleted BM cells were analyzed for LSK cell population. Data shown represent fold change over WT controls (n = 7). (**F**) Reduced absolute number of BM LSK cells in Pim TKO mice. The absolute LSK cells were calculated by multiplying the total number of BM cells (from 2 femurs and 2 tibias) with the percentage of LSK cells (n = 3, *p < 0.05 data representative of 3 independent experiments). (**G**) Unchanged percentage of LT-HSCs, ST-HSCs, and MPPs in Pim TKO mice. Bone marrow cells were harvested, depleted of RBCs and enriched for Lin^-^ cells using lineage depletion kit. The enriched Lin^-^ cells were then stained with CD34, CD135, Sca-1, and c-Kit antibodies as described in the Materials and Methods. Data represent fold changes over WT control mice (n=5).

Pim TKO mice were significantly smaller in size than heterozygous littermates [4]. Interestingly, when we measured the spleen weight and adjusted it to the total body weight, the Pim TKO mice still exhibited significant reduction in the spleen-to-body weight ratio. The spleen-to-body weight ratio was reduced by half in Pim TKO mice compared to sex- and age- matched WT controls (Figure 2A, p < 0.01). Additionally, Pim TKO mice had significantly lower number of total bone marrow cells (measured from 2 femurs and 2 tibias of each mouse) (Figure 2B, p < 0.05). Total number of splenocytes was also reduced in Pim TKO mice in comparison to WT controls (Figure 2C, p < 0.01). These data suggested a broadly diminished hematopoietic compartment in Pim TKO mice.

We next performed in vitro colony-forming unit (CFU) assay to determine the frequency of hematopoietic stem/progenitor cells (HSPCs) in the bone marrow (BM) of Pim TKO mice. As shown in Figure 2D, CFUs-granulocyte/macrophage (CFUs-GM), Burst-forming units-erythrocyte (BFUs-E), and CFUs-granulocyte, erythrocyte, monocyte, and megakaryocyte (CFUs-GEMM) were significantly reduced in Pim TKO mice, demonstrating reduced clonogenic activity of HSPCs in Pim TKO mice.

We also measured the percentage and absolute number of Lin^-^Sca-1^+^c-Kit^+^ (LSK) cells in BM (Figure 2E). LSK cells virtually account for all HSPCs in mice. We found that the percentage of LSK cells in the BM of Pim TKO mice was comparable to that in WT controls (Figure 2E). However, since the total BM cell mass was reduced in Pim TKO mice, the absolute number of LSK cells was significantly lower in Pim TKO mice than in WT mice (Figure 2F). To further characterize HSPC population in Pim TKO mice, we measured long-term (LT)-HSCs (CD34^-^CD135^-^ LSK cells), short-term (ST)-HSCs (CD34^-^CD135^+^ LSK cells), and multi-potential progenitor cells (MPPs; CD34^+^CD135^+^ LSK cells) [15]. We found that the percentages of LT-HSCs, ST-HSCs and MPPs were quite similar between Pim TKO mice and WT controls (Figure 2G), although one would expect that their absolute numbers would be decreased in Pim TKO mice.

### Reduced self-renewal and long-term repopulating capacity of HSCs in Pim TKO mice

**Figure 3:**
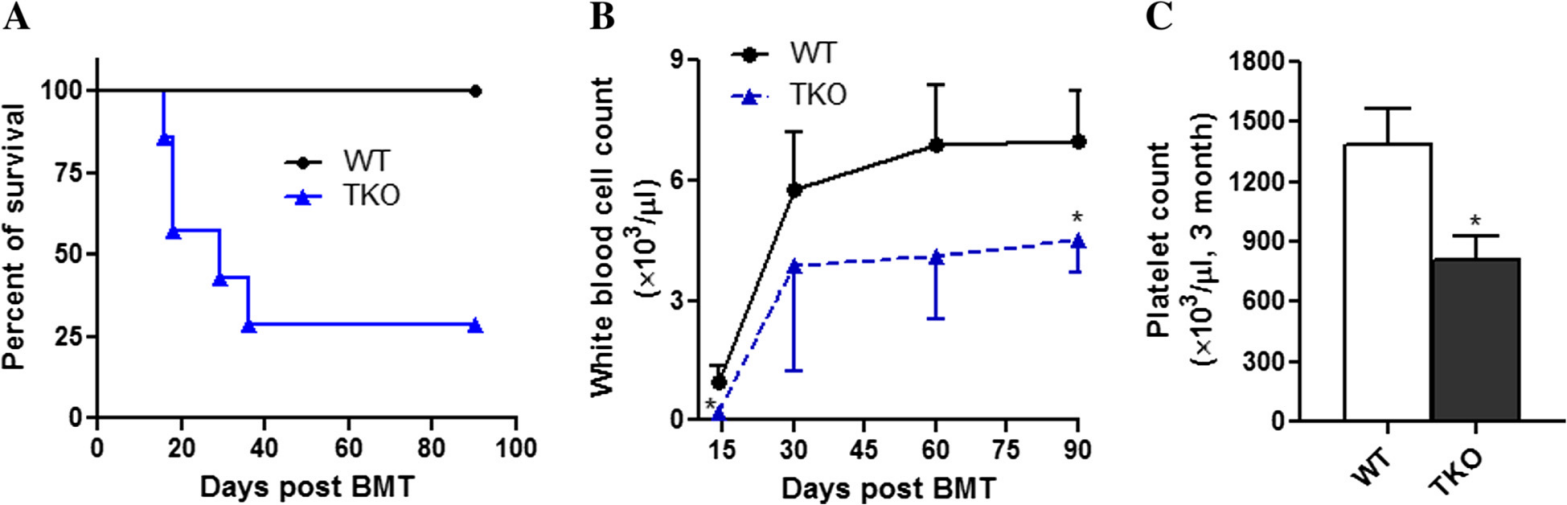
**Reduced animal survival and delayed hematological recovery in primary transplant mice receiving BM cells from Pim TKO mice.** Lethally irradiated female FVB/J mice were injected via tail vein with BM cells obtained from male Pim TKO mice or male WT controls (5 × 10^5^ cells/recipient mouse (n=7/group)). Animal survival (**A**) was monitored daily. Peripheral blood white blood cell count (**B**) was measured at 1, 2, and 3 months post transplantation (*p < 0.05). Peripheral platelet count (**C**) was measured at 3 months post transplant (*p < 0.05).

HSCs have the ability to self-renew and differentiate into all lineages of hematopoietic cells. These functions are best demonstrated using transplantation models in which the transplanted HSCs rescue lethally irradiated recipients and reconstitute the whole hematological system. We transplanted lethally irradiated (11 Gy) FVB/J mice with BM cells harvested from Pim TKO mice or sex- and age- matched WT controls (5×10^5^ cells/recipient mouse). As showed in Figure 3A, the mice transplanted with WT BM cells all survived after transplantation. In contrast, 75% of the mice transplanted with BM cells from Pim TKO mice died after transplantation. Additionally, those Pim TKO BM transplant recipient mice that survived showed significantly lower peripheral white blood cell counts at various time-points post transplantation (Figure 3B). The platelet count at 3 months post transplantation was also significantly lower in Pim TKO BM transplant recipient mice than that in the mice transplanted with WT BM cells (Figure 3C). The impaired hematological reconstitution seen in Pim TKO primary transplant recipient mice was not due to fewer number of LSK cells transplanted. All mice received equal number of BM cells and the percentages of LSK cells were similar between Pim TKO BM grafts and WT BM grafts (Figure 2E).

**Figure 4:**
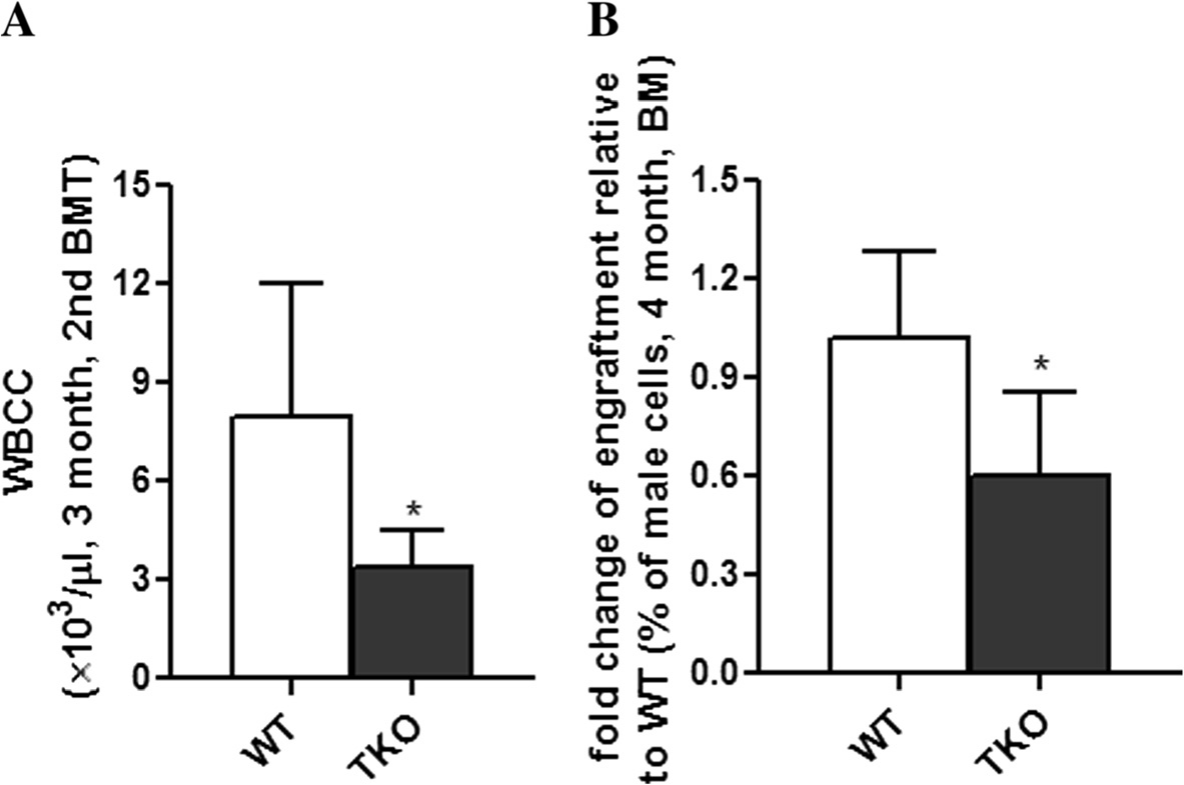
**Delayed hematological recovery and reduced donor cell contribution in secondary transplant mice receiving BM cells from Pim TKO primary transplant recipients.** BM cells were harvested from primary transplant recipients at 4 months post transplantation and injected into lethally irradiated female FVB recipients (1 × 10^7^/mouse). (**A**) Lower peripheral white blood cell counts in Pim TKO secondary transplant recipient mice. Peripheral white blood cell count in the secondary transplant recipient mice was measured at 3 months post transplant (n=6 for WT; n=3 for Pim TKO mice, *p < 0.05). (**B**) Reduced donor cell engraftment in the BM of Pim TKO secondary transplant recipient mice. The secondary transplant recipient mice were sacrificed at 4 months post transplant and BM cells were harvested and analyzed for male donor cell engraftment (*p < 0.05).

To further define the role of Pim kinases on HSC self-renewal and long-term repopulation, we performed secondary BM transplantation (Figure 4A-4B). BM cells were harvested from primary BM transplanted recipient mice at 4 months post transplantation and injected into lethally irradiated female FVB/J mice (1×10^7^ cells/mouse). At 3 months post transplantation, the secondary transplant mice receiving Pim TKO cells had significantly reduced peripheral white blood cell counts (Figure 4A). Donor cell engraftment in BM was quantified at 4 months post transplantation using a quantitative PCR-based method measuring the sex-determining region Y (Zfy1) [13,16]. We found that the male donor cells engraftment in secondary Pim TKO transplant recipients was significantly lower than that in controls (Figure 4B). Our data demonstrated that the self-renewal potential and the long-term repopulating capacity of HSCs were impaired in Pim TKO mice.

**Figure 5:**
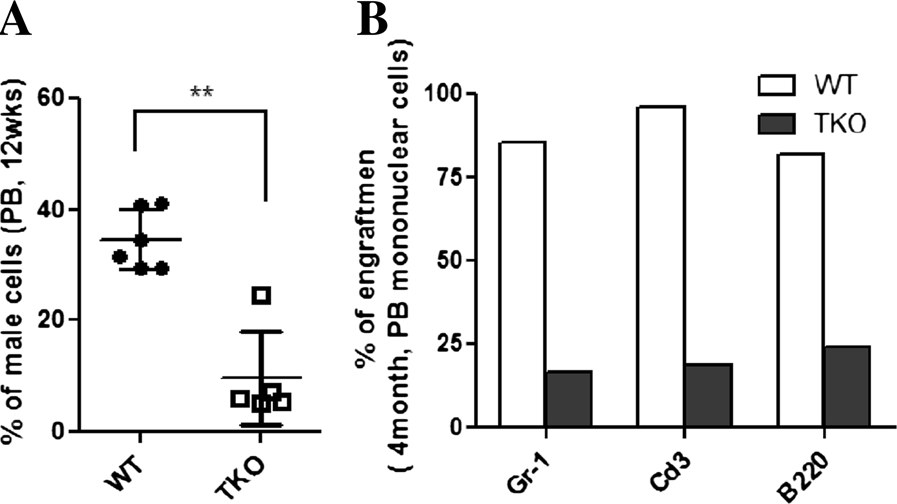
**Reduced repopulating capacity of Pim TKO BM cells in competitive transplants.** 5×10^5^ total BM cells from male Pim TKO mice or WT controls, along with 2×10^5^ female competitor BM cells, were transplanted into irradiated female recipients. (**A**) Reduced engraftment capacity of Pim TKO BM cells. Male donor engraftment in peripheral blood at 12 weeks post transplant was estimated (**p < 0.01). (**B)** Reduced engraftment in all three main cell subsets with Pim TKO BM cells. Gr-1^+^ granulocytes, CD3e^+^ T cells and B220^+^ B cells were sorted from pooled peripheral blood samples. Genomic DNAs from each population were isolated and the male donor cell contribution in these cell subsets was determined.

Competitive repopulating capacity of Pim TKO BM cells was also examined. In this series of experiments, 5×10^5^ BM cells from male Pim TKO mice or male WT controls were mixed with 2×10^5^ of competitive WT female FVB/J BM cells and transplanted into lethally irradiated female FVB/J recipients. Male donor cell engraftment in peripheral blood at 3 months post transplantation was estimated by quantitative PCR. As shown in Figure 5A, mice transplanted with Pim TKO BM cells had a lower percentage of donor- derived male cells compared to control mice (p < 0.01). These results indicated that Pim TKO BM cells were less efficient and less competent in reconstituting the hematopoietic system than WT BM cells. We also determined donor cell engraftment in three main peripheral blood cell subsets (i.e., Gr-1^+^ granulocytes, T cells and B cells) in the competitive transplant recipient mice. As shown in Figure 5B, the donor cell engraftment was reduced in all these three cell subsets in competitive transplant recipient mice receiving Pim TKO BM cells, suggesting impairment in multi-potential HSCs in Pim TKO mice.

### Reduced HSC proliferation and increased apoptosis in Pim TKO mice

**Figure 6:**
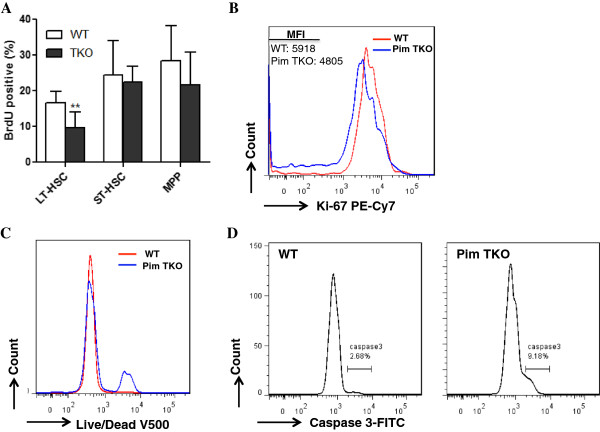
**Decreased proliferation and increased apoptosis of Pim TKO HSCs.** (**A**) Decreased in vivo BrdU incorporation in LT-HSCs of Pim TKO mice. Pim TKO mice or WT mice were injected with 2 doses of BrdU, and BM cells were harvested and labeled with antibodies. BrdU-positive cells in LT-HSCs, ST-HSCs, and MPPs were analyzed (n = 6, **P <0.01). (**B**) Decreased in vitro Ki67 labeling in Pim TKO LSK cells. LSK cells were sorted from Pim TKO mice or WT mice and cultured in StemSpan medium supplemented with TPO, SCF and Flt3 for 2 days. The cells were then stained with Ki67 antibody. Representative histogram of three independent experiments was shown (MFI: mean florescent intensity). (**C**) Increased cell death in Pim TKO LSK cells. The cultured LSK cells were stained with Live/Dead dye and representative histogram of three independent experiments was shown. (**D**) Increased caspase 3-activation in Pim TKO LSK cells. Cultured LSK cells were fixed and stained with caspase 3 antibody. Representative histogram of three independent experiments was shown.

To understand the mechanisms underlying the reduced capacity of Pim TKO HSCs in reconstituting hematopoiesis, we investigated the proliferation status of HSCs in Pim TKO mice. We first measured in vivo BrdU incorporation in LT-HSCs, ST-HSCs and MPPs of the mice. We observed significantly lower number of BrdU positive cells in the Pim TKO LT-HSC population (Figure 6A). The BrdU incorporation in ST-HSC and MPP compartments was not affected in Pim TKO mice (Figure 6A). We next measured Ki67 labeling in cultured LSK cells in vitro. We sorted BM LSK cells from Pim TKO mice or WT mice and cultured the cells in StemSpan medium supplemented with growth factors for 48 hours. Consistent with our in vivo BrdU results, we found that the mean fluorescent intensity (MFI) of Ki-67 expression in Pim TKO LSK cells was reduced by 20% compared to that in WT LSK cells (Figure 6B). These data suggested that Pim TKO HSCs were less proliferative.

To determine the role of Pim kinases in regulating HSC cell survival, we measured cell death and Caspase 3- activation in LSK cells ex vivo. Pim TKO LSK cells and WT LSK cells were cultured in vitro with growth factors for 48 hours. Cell death was measured by live/dead fixable dye and caspase 3- activation was determined by intracellular caspase 3 antibody staining. Compared to WT LSK cells, Pim TKO LSK cells showed increased rates of cell death (Figure 6C) and caspase 3- activation (Figure 6D).

## Discussion

Hematopoiesis is regulated by many different molecular pathways [17]. In the current study, we examined the role of Pim kinases in regulating the primitive HSCs in mice. We used serial transplant experiments, competitive transplant assay, and in vivo and in vitro proliferation assays to investigate the long-term repopulating HSCs in Pim TKO mice. Our study provides direct evidence for an important role of Pim kinases in hematopoiesis. Our findings are consistent with and support previous observations reported by others [4,10-12]. For instance, we found that Pim TKO mice had reduced body size, displayed erythrocyte microcytosis, and had reduced T cell numbers. We did not observe significant changes in the peripheral B- cell number. This is also consistent with previous observation by Mikkers, et al., who found that in young Pim TKO mice, peripheral B- cell numbers were reduced whereas in older animals, the B- cell number was unaltered [4]. Importantly, our current study extended our observations beyond previous results. We demonstrated that: 1). Deletion of Pim kinases affects multiple lineages of hematopoietic cells including platelet counts (Figures 1). 2). Deletion of Pim kinases affects the self-renewal and long-term repopulating capacity of HSCs (Figures 3, 4, 5). 3). Deletion of Pim kinases affects the proliferation of the most primitive HSCs in vivo and in vitro (Figure 6). and 4). Deletion of Pim kinases increases apoptotic cell death of HSCs (Figure 6). Our study provides new insights into the roles of Pim kinases in the regulation of HSCs.

The reasons the effects of Pim kinases in HSCs were not previously reported are: 1) Previous studies had been focused on the effects of Pim kinases on T- and B- cells. This seemed logical because overexpression of Pim1 kinase induced clonal T cell lymphoma/leukemia [8]. 2) Pim- deficient mice were generated on FVB/J background [4,10,11]. FVB/J mice lack readily available surface markers to separate donor-derived cells from congenic recipient-origin cells. This presents a technical challenge in determining the long-term repopulating capacity and self-renewal of HSCs in transplantation models. 3) The percentage of LSK cells in the BM of Pim TKO mice was comparable to that in WT controls, as we showed in Figure 2E. In the absence of serial transplant experiments, this finding may lead investigators to assume that HSC population is unaltered in Pim-deficient mice. The PCR-based method that we reported recently [13] allows us to reliably determine donor cell engraftment in our transplant experiments.

Several published studies suggested a potentially important role of Pim kinases in hematopoiesis and in HSCs. For example, Pim1 is highly expressed in human fetal hematopoietic tissues [18]. Additionally, Pim1 kinase is a key target for HOXA9, a homeoprotein important in hematopoiesis [19]. Pim1 and Pim3 were found to be important in maintaining the self-renewal of mouse embryonic stem cells, and loss of Pim1 and Pim3 led to cell differentiation [20]. Furthermore, overexpression of Pim kinase protected hematopoietic cells from apoptosis [21], and enhanced growth factor- independent survival in myeloid cells [22,23]. Recent study by Grundler, et al. [12] suggested that Pim1 kinase was critical in CXCR4 expression and HSC homing. Using transplant models, our study provides direct evidence for an important role of Pim kinases in hematopoiesis.

Pim kinases regulate diverse signal pathways in both hematological and non-hematological malignant cells. Pim kinases promote cell proliferation by regulating enzymes that are important in cell cycle progression, including Cdc25A [24,25] and p27kip1 [26]. Pim kinases regulate cell survival by phosphorylating the apoptotic protein BAD [27] and ASK1 [27]. Furthermore, Pim1 kinase regulates PRAS40 phosphorylation and increases the activities of mammalian target of rapamycin protein kinase [29]. Pim kinase was found to be important in controlling energy metabolism and cell growth [30]. Consistent with these observations in cancer cells, our studies suggest that Pim kinases are important in the regulation of cell proliferation and survival in HSCs.

Our study has important implications. Pim kinases are being investigated as a potential target in the drug development for the treatment of cancer [31]. Several compounds including pan- Pim inhibitors are currently under development and have shown interesting preclinical activities in multiple cancer histologies. Clinical data and safety profiles of these inhibitors in human are very limited. Our current studies suggest that it would be important to understand and monitor the potential hematological side effects when using Pim kinase inhibitors.

## Conclusions

We demonstrated that Pim kinases play a fundamental role in HSC regulation. Our findings support the notion that oncogenes are not only important in tumorigenesis, but also involved in normal cell development. Identifying these roles is an important step in developing safe and effective therapeutic agents for the treatment of cancers.

## Materials and methods

### Antibodies and reagents

APC-conjugated anti- mouse CD117 antibody (c-Kit, 2B8), APC-H7-conjuagted anti- mouse c-Kit antibody (2B8), PE- conjugated anti- mouse Sca-1 antibody (E13-161.7), APC-conjugated anti- mouse CD3e (145-2C11), PE-conjugated anti mouse Gr-1 (RB6-8C5), PE-Cy7-conjugated Ki67 antibody (B56), FITC- labeled Caspase3 (C92-605) antibody and FITC-BrdU Flow kit were purchased from BD Pharmingen (San Diego, CA). FITC- conjugated anti- mouse B220 (RA3-6B2); PerCP-eFluor 710- labeled anti- mouse CD135 (A2F10) and eFluor 450- conjugated anti- mouse CD34 (RAM34) antibodies were purchased from eBiosciences (San Diego, CA). Magnetic murine lineage cell depletion kit was purchased from Miltenyi Biotec (Auburn, CA). Aqua Live/dead fixable dye was purchased from Invitrogen (Grand Island, NY).

### Mice

#### Pim TKO mice

Pim1^−/−^2^−/−^ and Pim2^−/−^3^−/−^ double KO mice were generated by Mikkers, et al. [4] and were a kind gift of Drs Paul B. Rothman (Johns Hopkins University) and Anton Berns (The Netherlands Cancer Institute). Pim1^−/−^2^−/−^3^−/−^ TKO mice were generated by systematically breeding the Pim1^−/−^2^−/−^ and Pim2^−/−^3^−/−^ double KO mice and were on FVB/J background. Pim TKO mice and WT controls were maintained in our specific pathogen-free animal facility. The genotype of each mouse used in the study was confirmed by PCR genotyping of tail DNA.

#### FVB/J mice

WT FVB/J transplant recipient mice were purchased from the Jackson Laboratory. All our studies were performed in accordance with Medical University of South Carolina Institutional Animal Care and Use Committee approved- procedures.

### Peripheral blood cell subset analysis

Whole blood hematological parameters including white blood cell count, hemoglobin concentration, hematocrit, platelet count, mean corpuscular volume (MCV), and mean corpuscular hemoglobin (MCH) were measured using Scil ABC plus hematology analyzer (scil animal care company Ltd.) as per the manufacturer’s instructions. Peripheral blood cell subsets were quantified using flow cytometry as described previously [25]. Briefly, 50 μl blood was stained with monoclonal antibodies against various cell subsets [APC- CD3 (145-2C11), APC-Cy7- CD4 (GK1.5), PE-Cy7-CD8 (53–6.7), FITC- B220 (RA3-6B2), PE- Gr-1 (RB6-8C5)]. Equal volume (50 μl) of Flow-Count fluorospheres (Beckman-Coulter) was added before flow cytometric analysis. The absolute cell counts were calculated using the following formula: Absolute count (cells/μL blood) = (Total number of cells counted/Total number of fluorospheres counted) × Flow-Count fluorosphere concentration.

### Colony forming unit (CFU) assay

CFU assays were performed in complete M3434 methylcellulose medium (Stem Cell Technologies) following the manufacturer’s instructions. Briefly, BM cells from Pim TKO mice or WT controls were resuspended in complete M3434 medium and plated in 6-well plates at 1 × 10^5^ cells/well. The assays were done in triplicate and the number of CFUs-GM, BFUs-E and CFUs-GEMM was counted at day 7, day 9 and day 12, respectively.

### In vivo BrdU incorporation assay

In vivo BrdU incorporation was performed as described previously with minor modifications [32,33]. Briefly, mice were intraperitoneally injected with 2 doses (at 8 and 2 hours before sacrifice) of bromodeoxyuridine (5-bromo-2-deoxyuridine [BrdU]; BD Biosciences) at 50 μg/gram of body weight. BM cells were then isolated and enriched for Lin^-^ cell population using lineage cell depletion kit (Miltenyi Biotec). At least 1.5×10^6^ Lin^-^ BM cells were labeled with PE –conjugated anti- mouse Sca-1, APC- conjugated anti mouse c-Kit, PerCP-eFluor- conjugated anti mouse CD135, and eFluor 450- conjugated anti mouse CD34 antibodies, followed by fixation and staining with FITC–conjugated BrdU antibody (BrdU Flow Kit, BD Pharmingen), according to the manufacturer’s protocol.

### Hematopoietic stem cell transplantation (HCT)

For primary HCT, BM cells were isolated from male TKO mice or age matched WT controls. The red blood cell (RBC)- depleted BM cells were injected (cell doses were indicated in the text) via tail-vein to lethally irradiated (11Gy) female FVB/J recipient mice. Animal survival was monitored daily. To determine hematological recovery, peripheral blood was collected from transplant recipient mice by retro-orbital sampling under anesthesia condition. Whole blood cell counts were measured using a Scil ABC plus hematology analyzer as per the manufacturer’s instructions.

For secondary HCT, BM cells were obtained from primary transplanted recipient mice at 4 months post transplantation, and 1×10^7^ BM cells/recipient were injected into lethally irradiated female FVB/J mice. Male donor cell engraftment was measured.

For competitive repopulation assay, 5×10^5^ male BM donor cells from Pim TKO mice or WT controls were mixed with 2×10^5^ female competitive BM cells from FVB/J mice, and transplanted into lethally irradiated female FVB/J mice.

### Analysis of donor cell engraftment

Male donor cell engraftment in female transplant recipients was determined as described [13,16]. Briefly, genomic DNA was extracted from RBC- lysed peripheral blood cells or BM cells using the DNeasy Kit (QIAGEN), and further purified using Ethanol precipitation method. Twenty ng of genomic DNA were mixed with SYBR Green PCR master mix reagents (Bio-Rad) and real time PCR was performed. Donor cell engraftment was estimated by percentage of male DNA calculated from the standard curve by PCR for sex-determining region Y (Zfy1) [16]. Bcl-2: 5’-AAGCTGTCACAGAGGGGCTA and 5’-CAGGCTGGAAGGAGAAGATG or Actin: 5’-TGTTACCAACTGGGACGACA and 5’- ACCTGGGTCATCTTTTCACG were used as reference genes.

### Cell sorting for CD3e^+^ T cells, Gr-1^+^ granulocytes, and B220^+^ B cells

Peripheral blood samples (150 μl/mouse) were collected from each group (5 mice/group) at 4 months post competitive BM transplant and pooled. After RBC depletion, the leukocytes were stained with APC- conjugated anti mouse CD3e, PE –conjugated anti mouse Gr-1 and FITC -conjugated anti mouse B220 antibodies and subjected to cell sorting on Mo-Flo sorter (DakoCytomation). Sorted CD3e^+^ T cells, Gr-1^+^ granulocytes and B220^+^ B cells were processed for genomic DNA isolation. Male donor cell engraftment in each cell subset was determined by real time PCR analysis as described above.

### In vitro culture of LSK cells and cell proliferation and apoptosis assay

RBC-depleted total BM cells obtained from Pim TKO mice or WT control mice were first enriched for Lin^-^ cells by Lineage selection kit. Lin^-^ cells were then stained with Scal-1 and c-Kit antibodies and sorted on flow cytometry for LSK cells. LSK cells (6,000/well) were cultured in StemSpan SFEM medium (StemCell Technologies) supplemented with 100 ng/mL of murine stem cell factor (SCF), murine Thrombopoietin (TPO), and murine Flt3 (all from Invitrogen) for 2 days. Cells were then stained with Aqua Live/dead fixable dye (Invitrogen) followed by fixation and permeabilization (BD Cytofix/Cytoperm kit) according to the manufacturer’s instructions. The fixed cells were then stained with PE-Cy7-conjugated Ki-67 antibody and FITC-labeled caspase 3 antibody.

### Statistical analysis

The values were reported as Mean ± SEM of multiple experiments or Mean ± SD from a representative experiment. Differences were analyzed by Student’s t test. p < 0.05 was regarded as significant.

## Competing interests

The authors declare no competing financial interests.

## Authors’ contribution

NA performed research and analyzed data. ASK designed research. NA and YK designed research and wrote the paper. All authors reviewed and approved the final manuscript.

## Authors’ information

Ningfei An, PhD, is a postdoctoral research associate at the Medical University of South Carolina. Andrew Kraft, MD, is a professor of medicine and the director of the Hollings Cancer Center, Medical University of South Carolina. Yubin Kang, MD, is an assistant professor and a hematologist/oncologist at the Hollings Cancer Center, Medical University of South Carolina.
